# Supplementary material for: Genes ScBx1 and ScIgl—Competitors or Cooperators?
Source: Genes (Basel). 2020 Feb 20;11(2):223. doi: 10.3390/genes11020223 (PMC7074272; doi:10.3390/genes11020223)
Supplement: Supplementary file 1 [file genes-11-00223-s001.pdf]

Genes *ScBx1* and *ScIgl* – Competitors or Cooperators?

Anna Wlazło [1], Magdalena Święcicka [1], Marek D. Koter [1], Tomasz Krępski [1], Leszek Bolibok [2], Anna Stochmal [3], Mariusz Kowalczyk [3], Monika Rakoczy-Trojanowska [1,*]

[1] Department of Plant Genetics, Breeding and Biotechnology, Institute of Biology, Warsaw University of Life Sciences, 02-787 Warsaw, Poland; annawlazlo@outlook.com (A.W.); magdalena_swiecicka@sggw.pl (M.Ś.); marek_koter@sggw.pl (M.D.K.); tomasz_krepski@sggw.pl (T.K.); monika_rakoczy_trojanowska@sggw.pl (M.R.-T.)

[2] Department of Silviculture, Institute of Forest Sciences, Warsaw University of Life Sciences, 02-787 Warsaw, Poland; leszek_bolibok@sggw.pl (L.B.)

[3] Department of Biochemistry and Crop Quality, Institute of Soil Science and Plant Cultivation, State Research Institute, 24-100 Puławy, Poland; asf@iung.pulawy.pl (A.S.); mkowalczyk@iung.pulawy.pl (M.K.)

* Correspondence: monika_rakoczy_trojanowska@sggw.pl; Tel.: +48-225932150

ORCID: 0000-0001-6891-0471

Table S1. The content of BX in plants of three inbred lines, L318, D33, and D39 measured after the period of natural vernalization [16].

Line	BX content [µg/mg d.m.]					
	HBOA	GDIBOA	DIBOA	GDIMBOA	DIMBOA	MBOA
L318	0.01297	0.02594	1.17881	0	0	0
D33	0.0153	0.1736	0.84587	0.0019	0	0.0015
D39	0.0099	0.1755	0.4760	0.00003	0	0.0012

Table S2. Parameters of mass spectrometry analysis.

Compound	RT [min]	*m*/*z* of the parent $[M-H]^-$ ion	*m*/*z* of fragment ions		Collision energy [eV]	Cone voltage [V]
IbG (IS)	3.70	294	quantifier	131	15	20
			qualifier	174		
HBOA	4.15	164	quantifier	108	15	30
			qualifier	136		
DIBOA	4.45	180	quantifier	134	6	20
			qualifier	124		
GDIBOA	5.10	342	quantifier	162	15	25
			qualifier	180		
DIMBOA	6.30	210	quantifier	149	6	15
			qualifier	195		
GDIMBOA	6.70	372	quantifier	149	15	35
			qualifier	210		
MBOA	8.00	164	quantifier	149	15	20
			qualifier	121		

Table S3. The values of r coefficients for correlations between the expression level of the genes *ScBx1* and *ScIgl*, and the content of BXs (based on **Spearman's rho**).

IL	BX / gene	I *ScBx1*	I *ScIgl*	II *ScBx1*	II *ScIgl*	III *ScBx1*	III *ScIgl*	IV *ScBx1*	IV *ScIgl*	V *ScBx1*	V *ScIgl*	VI *ScBx1*	VI *ScIgl*
L318	HBOA	0.83**	0.00	-0.10	-0.40	0.00	0.63	anpo	0.65	anpo	0.50	anpo	-0.03
	GDIBOA	-0.77	0.54	0.09	0.46	-0.58	0.29	anpo	0.41	anpo	0.52	anpo	-0.03
	DIBOA	0.6	-0.14	0.77	-0.87**	-0.93**	0.7	anpo	0.5	anpo	0.44	anpo	0.03
	GDIMBOA	-0.77	0.54	0.54	-0.52	-0.52	-0.35	anpo	anpe	anpo	0.46	anpo	0.17
	DIMBOA	-0.75	-0.23	0.6	-0.46	-0.52	-0.23	anpo	anpe	anpo	0.46	anpo	-0.03
	MBOA	-0.75	-0.23	0.43	-0.84**	-0.93**	0.46	anpo	anpe	anpo	0.52	anpo	0.4
D33	HBOA	-0.81	0.62	-0.65	-0.65	-0.39	0.39	0.44	-0.50	anpo	0.52	anpo	-0.49
	GDIBOA	0.46	-0.03	-0.70	-0.67	-0.93**	0.70	-0.93**	0.99**	anpo	0.52	anpo	-0.60
	DIBOA	0.52	-0.77	-0.94**	-0.43	-0.43	0.09	-0.46	0.52	anpo	-0.41	anpo	-0.60
	GDIMBOA	0.06	-0.60	-0.43	-0.94**	-0.94**	0.71	-0.43	0.43	anpo	-0.06	anpo	0.14
	DIMBOA	0.52	-0.60	-0.94**	-0.43	-0.49	-0.09	0.37	-0.49	anpo	-0.50	anpo	-0.89**
	MBOA	-0.46	0.83**	-0.26	-0.94**	-0.43	0.09	-0.03	-0.03	anpo	0.52	anpo	-0.54
D39	HBOA	-0.50	-0.97**	anpe	anpe	anpo	anpe	anpo	anpe	anpo	1.00	anpo	-0.46
	GDIBOA	-0.46	-0.99**	0.87**	-0.49	anpo	0.46	anpo	-0.89**	anpo	0.54	anpo	-0.43
	DIBOA	-0.37	-0.94**	-0.93**	0.70	anpo	0.41	anpo	-0.37	anpo	1.00**	anpo	0.37
	GDIMBOA	0.43	0.89**	0.37	0.26	anpo	0.93**	anpo	-0.49	anpo	0.43	anpo	-0.77
	DIMBOA	0.85**	0.78	-0.93**	0.70	anpo	anpe	anpo	anpe	anpo	0.09	anpo	-0.03
	MBOA	0.09	-0.03	-0.31	0.54	anpo	anpe	anpo	anpe	anpo	anpe	anpo	-0.94**

* – significant at $p < 0.1$

** – significant at $p < 0.05$

anpo - analysis not possible because of undetectable expression of *ScBx1* gene

anpe - analysis not performed because of the equal values of BX contents

Table S4. Proteins bound to the promoter sequences of the *ScBx1* and *ScIgl* genes annotated in the *in vivo* assay.

ScBx1 Prs-treated	*ScIgl Prs*-treated	*ScBx1* untreated	*ScIgl* untreated
HMG-I/Y protein (HMGa) [*Triticum aestivum*] AAM22691.1	Methyl-CpG-binding domain-containing protein 9 (MBD9); PKA61759.1	HMG-I/Y protein (HMGa); AAM22691.1	**NAC domain containing transcription factor (NAC042); ANR02348.1**
**germin-like protein (GLP1) AEN02469.1**	**germin-like protein (GLP1); AEN02469.1**	KIX_2 domain-containing protein; ABK26292.1	Integrase catalytic domain-containing protein (TCM_012637); EOY21236.1
histone deacetylase 2 (HDAC2); ABG43091.1	Integrase catalytic domain-containing protein (TCM_012637); EOY21236.1	HMG-Y-related protein A (TRIUR3_33243); EMS65335.1	**Zinc finger protein (ZAT1_1); PWZ38869.1**
KIX_2 domain-containing protein; ABK26292.1	hypothetical protein (CFOL_v3_04877), partial; GAV61350.1	Integrase catalytic domain-containing protein (TCM_012637); EOY21236.1	PREDICTED: uncharacterized protein (LOC103500952) isoform X2; XP_008462641.1
H15 domain-containing protein; AFK35979.1	NusB domain-containing protein (Ahy_B04g070543) isoform B; RYR13675.1	DNA/RNA helicase, DEAD/DEAH box type, N-terminal (Ccrd_016929); KVI04750.1	**RING-H2 finger protein ATL8-like; XP_020242355.1**
predicted protein BAK00355.1	**protein EXORDIUM-like 2; XP_002960721.1**	**protein EXORDIUM-like 2; XP_002960721.1**	LOW QUALITY PROTEIN: DNA-directed RNA polymerase 3B, chloroplastic; XP_020980201.1
unnamed protein product (TRAES_3BF060500360CFD_c1); CDM80146.1	PREDICTED: uncharacterized protein (LOC103500952) isoform X2; XP_008462641.1	PREDICTED: pre-mRNA-splicing factor ATP-dependent RNA helicase (PRP16); XP_004148974.1	
Integrase catalytic domain-containing protein (TCM_012637); EOY21236.1	PREDICTED: sister chromatid cohesion protein (PDS5) homolog A-like isoform X1; XP_011040159.1	PREDICTED: serine/arginine-rich splicing factor RSZ22-like; XP_008337095.1	
HMG1/2-like protein; P40621.1	LOW QUALITY PROTEIN: DNA-directed RNA polymerase 3B, chloroplastic; XP_020980201.1	PREDICTED: uncharacterized protein (LOC103500952) isoform X2; XP_008462641.1	
MYND-type domain-containing protein (CHLRE_17g740000v5) PNW70945.1		PREDICTED: sister chromatid cohesion protein (PDS5) homolog A-like isoform X1; XP_011040159.1	
**NIM1-interacting TFIIH subunit (TorRG33x02_009350);** POO02874.1		**RING-H2 finger protein ATL8-like; XP_020242355.1**	
TOP4c domain-containing protein (GQ55_9G036200); PUZ36416.1		LOW QUALITY PROTEIN: DNA-directed RNA polymerase 3B, chloroplastic; XP_020980201.1	
**Zinc finger protein (ZAT1_1); PWZ38869.1**		structural maintenance of chromosomes protein 5-like (PHYPA_028643); XP_024364315.1	
Nucleosome assembly protein 1;3-like (C5167_004537), partial; RZC57230.1		**agamous-like MADS-box protein (AGL62); XP_027364274.1**	
PREDICTED: uncharacterized protein (LOC103500952) isoform X2; XP_008462641.1			
DNA repair protein RAD5B; XP_010918825.1			
sister chromatid cohesion protein (PDS5) homolog A; XP_021902104.1			
SpoU_methylase domain-containing protein (LOC112275201); XP_024361104.1			
**probable transcription factor (KAN4) myb-like DNA-binding domain; XP_026398713.1**			
**agamous-like MADS-box protein (AGL62); XP_027364274.1**			
No. of proteins bound to the promoter sequences			
20	9	14	6

Prs - *Puccinia recondita* f.sp. *secalis* (Roberge ex Desmaz),
stress related transcription factors (TFs) are marked in bold
growth and development related TFs are marked in bold and underlined by a single line

stress related and growth and development related TFs are marked in bold and underlined by a dotted line

Table S5. Normalized expression level of *ScBx1* and *ScIgl* genes in leaves of rye cv. Stach F1 inoculated with BSMV:*ScBx1* on the 14th dpi.

Plant	Normalized expression level of *ScBx1*	Normalized expression level of *ScIgl*
# **1**	0.00	0.89
# **3**	0.06	1.16
# **5**	0.06	0.21
# **7**	0.00	1.47
# **9**	0.02	6.26

The relative expression level of *ScBx1* and *ScIgl* gene was normalized to their expression level in plants inoculated with BSMV:α,$\beta_{(-)}$,$\gamma_{(PDS)}$ vector assumed as 1.

Table S6. Normalized expression level of *ScBx1* and *ScIgl* genes in leaves of rye cv. Stach F1 inoculated with BSMV:*ScBx1* on the 21th dpi.

Plant	Normalized expression level of *ScBx1*	Normalized expression level of *ScIgl*
# **2**	0.00	0.31
# **5**	0.00	0.14
# **6**	0.01	0.13
# **8**	0.02	0.74
# **10**	0.05	0.32
# **11**	0.00	0.32
# **12**	0.03	0.98
# **13**	0.00	1.89
# **14**	0.12	1.15

The relative expression level of *ScBx1* and *ScIgl* gene was normalized to their expression level in plants inoculated with BSMV:α,$\beta_{(-)}$,$\gamma_{(PDS)}$ vector assumed as 1.
